# Dietary Nitrate from Plant Foods: A Conditionally Essential Nutrient for Cardiovascular Health

**DOI:** 10.1016/j.advnut.2023.100158

**Published:** 2023-11-24

**Authors:** Ana Clara da C Pinaffi-Langley, Rosa M Dajani, M Catherine Prater, Hoang Van M Nguyen, Kurt Vrancken, Franklin A. Hays, Norman G Hord

**Affiliations:** 1 Department of Nutritional Sciences, College of Allied Health, University of Oklahoma Health Sciences Center, Oklahoma City, OK, United States; 2 Nutrition and Food Services, San Francisco Health, University of California, San Francisco, CA, United States; 3 Department of Foods and Nutrition, Dawson Hall, University of Georgia, Athens, GA, United States; 4 Independent Researcher, Brooklyn, NY, United States; 5 Department of Nutritional Sciences, College of Education and Human Sciences, Oklahoma State University, Stillwater, OK, United States

**Keywords:** nitrate, nitric oxide, nitric oxide synthase, nitrite, vasodilation, gasotransmitter

## Abstract

Under specific conditions, such as catabolic stress or systemic inflammation, endogenous nutrient production becomes insufficient and exogenous supplementation (for example, through dietary intake) is required. Herein, we propose consideration of a dietary nitrate from plant foods as a conditionally essential nutrient for cardiovascular health based on its role in nitric oxide homeostasis. Nitrate derived from plant foods may function as a conditionally essential nutrient, whereas nitrate obtained from other dietary sources, such as drinking water and cured/processed meats, warrants separate consideration because of the associated health risks. We have surveyed the literature and summarized epidemiological evidence regarding the effect of dietary nitrate on cardiovascular disease and risk factors. Meta-analyses and population-based observational studies have consistently demonstrated an inverse association of dietary nitrate with blood pressure and cardiovascular disease outcomes. Considering the available evidence, we suggest 2 different approaches to providing dietary guidance on nitrate from plant-based dietary sources as a nutrient: the Dietary Reference Intakes developed by the National Academies of Sciences, Engineering, and Medicine, and the dietary guidelines evaluated by the Academy of Nutrition and Dietetics. Ultimately, this proposal underscores the need for food-based dietary guidelines to capture the complex and context-dependent relationships between nutrients, particularly dietary nitrate, and health.

**Statement of significance**

Overwhelming evidence supports the consideration of dietary nitrate from plant foods as a conditionally essential nutrient for cardiovascular health based on its role in nitric oxide homeostasis, which may contribute to population health.

## Introduction

Standards for essential nutrients, such as the Dietary Reference Intake (DRI) [1], are established for healthy individuals within populations classified according to age group and sex. The DRIs are nutrient reference values that serve as the scientific basis for food guidance in the United States and Canada including many program, policy, and regulatory initiatives. Conditionally essential nutrients, to the contrary, refer to certain nutrients whose endogenous production cannot meet physiological needs in conditions such as neonatal growth, catabolic stress, or disease conditions [2], requiring exogenous supplementation (for example, through diet). In this context, standards for conditionally essential nutrients are referred to as Specialized Nutrient Requirements [2,3]. Examples of conditionally essential nutrients include choline, glutamine, and arginine. More recently, other compounds such as creatine [4] have been proposed as conditionally essential nutrients.

In this Perspective article, we continue to make the case [[5], [6], [7], [8]] for the consideration of nitrate from plant foods as a conditionally essential nutrient for human health based on its role in nitric oxide (NO) homeostasis. We aimed to inform potential future dietary recommendations for the consumption of plant-based food sources of nitrate based on literature data regarding health benefits and risks. Finally, we highlighted the factors that can affect nitrate bioavailability and bioaccessibility that should be addressed in future studies aiming to elucidate potential health benefits and risks associated with dietary nitrate intake.

## Nitric oxide physiology and function

NO, an amphipathic free-radical molecule, was identified as the elusive endothelium-derived relaxing factor in the 1980s [9]. Depending on physiological context, NO can act as an agonist of soluble guanylate cyclase or signal via nitrosylation and/or nitration of organic molecules (for example, sulfur-containing amino acids and unsaturated fatty acids) to generate paracrine or endocrine effectors. These effectors modulate immune, metabolic, and platelet function; vasodilation; and neurotransmitter release (for recent exhaustive reviews on NO signaling and physiology, see Lundberg and Weitzberg [10] and Kapil et al. [11]). Importantly, we recognize that the physiological functions of NO are diverse and context-specific. With that, herein we focused on physiological effects on the cardiovascular system as a basis for our recommendations because the most comprehensive body of evidence relating dietary nitrate and nitrite to health effects is related to cardiovascular disease and associated risk factors.

Physiological manifestations of NO deficiency are evident from human studies linking impaired NO bioavailability to increased risk of inflammation, atherosclerosis, and mortality [[12], [13], [14]]. Under conditions of exacerbated physiological stress such as chronic inflammation, the enzyme responsible for endogenous NO production in endothelial cells, endothelial nitric oxide synthase (eNOS), may generate reactive oxygen species–particularly superoxide anion ($O_{2}^{∙-}$)–instead of NO [15] and thus decrease NO production. This change in enzymology is termed eNOS uncoupling. Oxidative stress is a main cause of eNOS uncoupling, creating a feedback loop that increases reactive oxygen species generation, increases peroxynitrite (ONOO^-^) formation from NO and superoxide anion, and decreases NO bioavailability to the endothelium. In particular, eNOS uncoupling and impaired NO bioavailability result in endothelial dysfunction, a seminal pathophysiological feature of atherosclerosis and various cardiovascular diseases [16].

Studies have reported a decrease in whole-body NO synthesis rate of 31%, 61%, 73%, and 40% in patients with hypertension [17], chronic renal failure [18], chronic heart disease [19], and hypercholesterolemia [12], respectively, compared with that of their healthy counterparts. Furthermore, Kleinbongard et al. [13] reported that plasma nitrite concentration—a biomarker of NO bioavailability—decreased significantly with an increase in cardiovascular disease risk factors. They also reported that plasma nitrite levels were inversely correlated with the degree of endothelial dysfunction as measured by flow-mediated dilation and intima media thickness [13]. These findings suggest that pathophysiological sequelae resulting from NO deficiency may lead to sustained oxidative stress, atherosclerosis, and other cardiometabolic diseases, emphasizing the importance of NO homeostasis in various physiological systems. It is in this context that we describe the NO axis, a highly dynamic and interconnected system of NO production exhibiting functional redundancy. Different physiological conditions, especially those associated with pathophysiological processes such as inflammation present in certain disease states, as well as the complex biochemistry of distinct biological compartments, dictate the metabolic disposition of these pathways. As such, the NO axis responds to endogenous (NOS-dependent NO production) and exogenous (dietary nitrate and nitrite; organic nitrates such as nitroglycerin) inputs, local redox conditions, and local oxygen tension [20].

## Endogenous nitric oxide production

The discovery of endogenous NO synthesis and its expansive role in physiology has challenged the understanding of cellular communication and inaugurated the concept of endogenous gasotransmitters, which currently include NO, hydrogen sulfide, carbon monoxide, sulfur dioxide, and cyanide [[21], [22], [23]]. In humans, NO is generated endogenously using L-arginine, molecular oxygen, tetrahydrobiopterin, and NADPH. This reaction is catalyzed by a specific enzyme, nitric oxide synthase (NOS), which exists in 3 isoforms: endothelial (eNOS), neuronal (nNOS), and inducible (iNOS) [24]. For a detailed review on the mechanistic aspects of NOS, which is beyond the scope of this article, we refer the reader to seminal publications in the field [[25], [26], [27]]. In addition to NOS-mediated NO production, recent findings uncovered a physiological role for generation of NO from exogenous substrates, namely nitrate and nitrite, that are described in the next section.

## Exogenous contribution to nitric oxide production: the nitrate–nitrite–nitric oxide pathway

**FIGURE 1:**
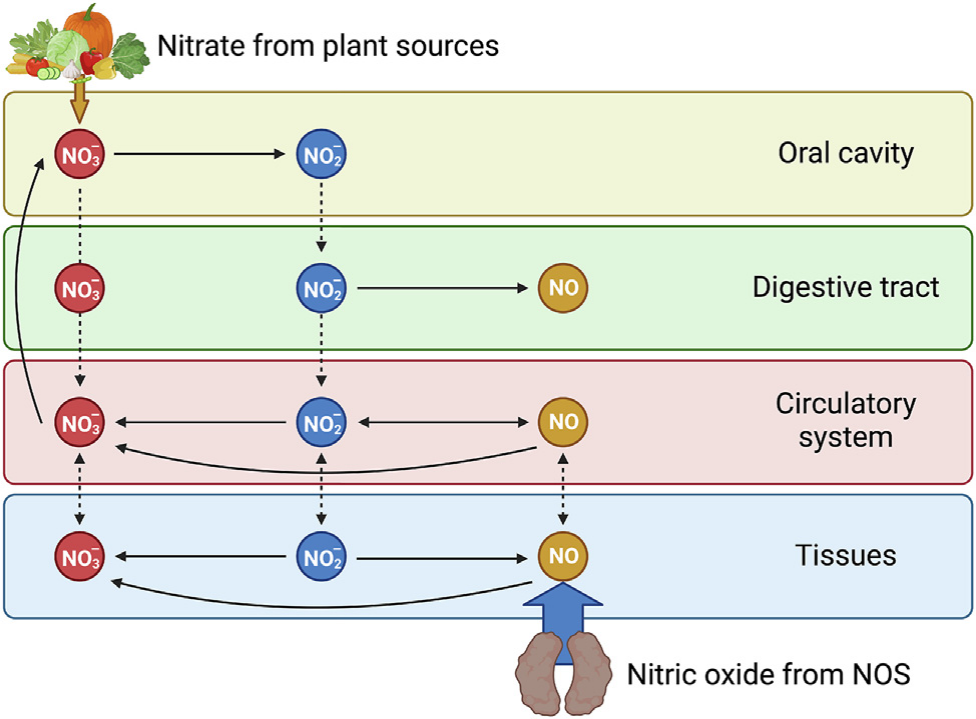
Nitric oxide (NO) axis with inputs from nitric oxide synthase (NOS)-dependent and -independent pathways. Dietary nitrate is readily absorbed within the digestive tract and distributed throughout the body. Circulating nitrate absorbed from dietary sources as well as nitrate resulting from NOS-generated NO oxidation enters the enterosalivary circulation and are concentrated in the saliva. In the oral cavity, commensal bacteria residing on the tongue’s surface reduce nitrate to nitrite, which is then swallowed and reaches the stomach. In the stomach, nitrite is partly reduced to NO via acidic disproportionation. Nitrite that is not reduced in the stomach is reduced to NO in the blood and tissues by various proteins with nitrite-reducing capacity (e.g., xanthine oxidoreductase, aldehyde oxidase, deoxyhemoglobin, deoxymyoglobin). On the other side of the NO axis, the majority of the body’s NO originates from NOS within the endothelium. Close to its site of origin, the highly reactive NO radical can be rapidly oxidized to nitrite. Both nitrite and NO can be readily oxidized to nitrate within the circulation and tissues. Excess nitrate is excreted through the kidneys. Created with BioRender.com.

The recently characterized nitrate–nitrite–NO pathway is an important complementary system for NO production [28]. In mammals, estimates indicate that 70% of plasma nitrite is produced via an eNOS-dependent pathway [29]. In turn, the NOS-independent nitrate–nitrite–nitric oxide pathway produces the remainder of NO present in the body [10]. This pathway involves the sequential reduction of nitrate to nitrite, and nitrite to NO. These mechanisms are highly context-dependent and occur within specific biological compartments and biochemical circumstances. In the nitrate–nitrite–nitric oxide pathway, circulating nitrate absorbed from dietary sources as well as nitrate resulting from NOS-generated NO oxidation enters the enterosalivary circulation and is actively accumulated in saliva, where its concentration reaches 10-fold higher than that found in plasma [30]. Within the oral cavity, commensal bacteria residing on the tongue reduce nitrate to nitrite, an essential step for the bioactivation of nitrate and critical for systemic NO homeostasis [31]. Nitrite then reaches the stomach, where it is partly reduced to NO via acidic disproportionation. In this compartment, dietary components such as polyphenols, cocoa flavanols, and vitamin C can mediate this non-enzymatic reduction [[32], [33], [34]]. Moreover, other biologically active nitrogen oxides generated in the stomach undergo secondary nitrosating and nitrating reactions with low molecular weight thiols, heme centers, proteins, ethanol, and unsaturated fatty acids [35] to generate products such as S-nitrosylation of vascular protein kinase C [36], nitro-oleic acid [37,38], and ethylnitrite [39]. Nitrite that escapes reduction in the stomach enters the circulation and is reduced to NO in the blood and tissues by various intermediaries (for example, xanthine oxidoreductase, aldehyde oxidase, deoxyhemoglobin, deoxymyoglobin) [40] (Figure 1).

Seminal discoveries over the past 50 y helped characterize the nitrate–nitrite–NO pathway described above and its role in NO homeostasis [28]. Milestones in establishing this new pathway include the demonstration that dietary nitrate increases salivary [30] and plasma nitrite [41], and that plasma nitrite serves as a substrate for NO production [42,43]. In 1997, Duncan and collaborators [44] reported that enterosalivary nitrate circulation is crucial to enable oral nitrate reduction to nitrite by lingual bacteria. This observation is strengthened by subsequent discoveries indicating that nitrate is associated with blood pressure-lowering effects via plasma nitrite and/or S-nitrosothiols and/or red blood cell cyclic GMP release [45], and that antiseptic mouthwash abolishes this effect by preventing bacterial nitrate reduction [46,47]. Recently, Yang et al. [48] demonstrated that exogenous nitrate increases cyclic GMP release from erythrocytes, thus leading to cardioprotective effects. Taken together, these studies established a new pathway for NO production that is favored by conditions under which NOS-dependent pathways are impaired, namely, hypoxic and acidotic conditions.

Pre-clinical and epidemiological studies associating low nitrate intake with adverse health effects provide further evidence of the crosstalk between NOS-dependent and -independent pathways. For instance, nitrate-free diets in a rodent model led to hyperglycemia, adiposity, and premature death because of cardiovascular disease [49]. Pathological features of NO deficiency associated with low nitrate intake are also seen in diabetes [50], poor muscle function [[51], [52], [53], [54], [55]], eye function and age-related macular degeneration [[56], [57], [58], [59]], and cognition and cerebrovascular health [[60], [61], [62], [63]]. Furthermore, in conditions of insufficient vascular NO production, intervention with inorganic nitrate or nitrite can rescue phenotypes such as hypertension that are induced by NOS-dependent pathway dysfunction, including rodent eNOS knockout models and human inherited arginosuccinate lyase deficiency [[64], [65], [66], [67]].

Conversely, conditions of excessive systemic NO availability are observed with chronic intake of organic nitrate pharmaceuticals such as nitroglycerin and long-term high oral nitrate or nitrite exposures [68,69]. Pharmacologic agents such as nitroglycerin are prescribed for vasodilatory and anti-ischemic effects in the treatment of angina pectoris and heart failure [70]. However, chronic intake of organic nitrates leads to a phenomenon called nitrate tolerance, defined as an attenuation or loss of the hemodynamic and anti-ischemic effects of these drugs, a well-recognized clinical problem that limits their clinical utility. Tolerance to organic nitrates may, in part, be caused by eNOS uncoupling through multiple mechanisms including oxidative depletion of tetrahydrobiopterin, oxidative disruption of the eNOS dimer, inhibitory phosphorylation of eNOS at threonine or tyrosine residues, redox-triggered accumulation of asymmetric dimethylarginine, and L-arginine deficiency [71]. This is one example whereby pharmacological provision of excess NO can lead to pathophysiological consequences.

Dietary exposure to nitrate at normal dietary concentrations in human and animal studies has salutary effects on blood pressure [72,73]. In contrast, excessive intake representative of pharmacological doses of nitrate is associated with hypertensive effects in rodents [68]. In humans, long-term (>10 wk) nitrite supplementation at pharmacological doses abolished the initial antihypertensive effect of the intervention, indicating a tolerance mechanism [69]. Thus, as with many essential nutrients, such as zinc [74], dietary deficiency or excess represents a U-shaped dose-response curve regarding adverse health effects. Taken together, this evidence suggests that, in the context of NOS dysfunction, exogenous input of optimal/sufficient concentrations of dietary nitrate and/or nitrite into the NO axis becomes critical for maintaining NO homeostasis. Thus, nitrate intake from plant foods at dietary rather than pharmacological concentrations may exert functions as a conditionally essential nutrient to address physiological sequelae associated with low NO bioavailability.

## Dietary nitrate as a conditionally essential nutrient: focus on cardiovascular health

Dietary nitrate has potential to act as a conditionally essential nutrient in conditions that involve low dietary nitrate, decreased NOS expression, dysfunctional NOS activity, and/or increased NO auto-oxidation, which all ultimately result in decreased NO bioavailability [[12], [13], [14],^16^]. The 3 main dietary nitrate exposure sources are plant foods (especially leafy green and root vegetables), drinking water, and animal-based foods (especially cured and processed meats). In this section, we are focusing on plant food sources of nitrate for their potential role as a conditionally essential nutrient. Potential health risks associated with the other exposure routes are addressed in the following section.

The largest body of evidence supporting the role of dietary nitrate in human health focuses on cardiovascular diseases and associated risk factors, especially hypertension. As described previously, these conditions are associated with impaired NO synthesis rate and decreased plasma nitrite levels, which indicate loss of NO homeostasis [12,13,[17], [18], [19],^29^]. Since the discovery of the nitrate–nitrite–NO pathway, nitrate supplementation has emerged as a practical strategy to enhance systemic NO bioavailability. In addition to the widely explored effect of inorganic nitrate on vasodilation and blood pressure [75], studies have also investigated other mechanisms through which dietary nitrate can promote cardiovascular health. These nitrate-mediated mechanisms include endothelial function improvement [46,76], sympathetic outflow modulation [77,78], NADPH oxidase activity inhibition [79,80], angiotensin II receptor signaling modulation [81], arterial stiffness reduction [82], platelet aggregation inhibition [83], soluble guanylate cyclase activation and cyclic GMP release [48], and atherosclerotic plaque stabilization [84]. Taken together, these findings demonstrate that dietary nitrate can act through pleiotropic mechanisms to reduce atherosclerotic cardiovascular disease risk.

Several meta-analyses have consistently shown an inverse association between main dietary sources of plant-based nitrate–green leafy vegetables–and cardiovascular disease and type 2 diabetes mellitus risk [[85], [86], [87], [88], [89], [90]]. A recently published study using National Health and Nutrition Examination Survey data found that urinary nitrate, a frequently used indicator of nitrate intake in humans, is positively associated with lower risk of congestive heart failure; diabetic nephropathy; and all-cause, cardiovascular disease, and diabetes mortalities [14]. Furthermore, population-based observational studies have highlighted inverse associations between dietary nitrate intake on long-term cardiovascular disease outcomes and all-cause mortality (Table 1) [[91], [92], [93], [94], [95]]. Collectively, these studies collected data on usual dietary nitrate intake for over 100,000 people with an average follow-up length of 17 y.

**TABLE 1:** Study characteristics and results of population-based observational studies on the association between dietary nitrate intake and cardiovascular disease outcomes

First author	Year	Number of participants	Population/cohort	Follow-up length	Comparator	Outcome	Results
Z. Bahadoran [91]	2016	2799	Adults (≥20 y old) Tehran Lipid and Glucose Study	5.8 y	Lowest (<6.04 mg/d) vs. highest (≥12.7 mg/d) tertile	Incident HTN and CKD	Dietary nitrate: no association with HTN or CKD Dietary nitrite: OR 0.58 (95% CI: 0.33, 0.98) for HTN; OR 0.50 (95% CI: 0.24, 0.89) for CKD
L. Blekkenhorst [92]	2017	1226	Older adults (70–85 y old)	15 y	1-SD increase from the mean (67.0 ± 29.2 mg/d)	ASVD and all-cause mortality	ASVD mortality: HR 0.79 (95% CI: 0.68, 0.93; *P* = 0.004) All-cause mortality: HR 0.87 (95% CI: 0.78, 0.97; *P* = 0.011)
J. Jackson [93]	2019	62,535	Female adults (30–55 y old) Nurses’ Health Study	26 y	Lowest (<86 mg/d) vs. highest (>195 mg/d) quintile	CHD risk	RR 0.91 (95% CI: 0.80, 1.04; *P* = 0.27)
A. Liu [94]	2019	2229	Adults (≥49 y old) Blue Mountains Eye Study	14 y	Lowest (<69.5 mg/d) vs. highest (>137.8 mg) quartile	CVD mortality	HR 0.63 (95% CI: 0.41, 0.95)
C. Bondonno [95]	2021	53,150	Adults (≥49 y old) Danish Diet, Cancer, and Health Study	23 y	Lowest (median intake: 23 mg/d) vs. highest (median intake: 141 mg/d) quintile	BP and CVD risk	SBP: −2.58 mmHg (95% CI: −3.12 to −2.05 mmHg) DBP: −1.38 mmHg (95% CI: −1.66 to −1.10 mmHg) CVD risk: HR 0.86 (95% CI: 0.82, 0.91)

Abbreviations: ASVD, atherosclerotic vascular disease; BP, blood pressure; CI, confidence interval; CKD, chronic kidney disease; CVD, cardiovascular disease; DBP, diastolic blood pressure; HR, hazard ratio; HTN, hypertension; RR, risk ratio; SBP, systolic blood pressure.

However, it is important to highlight the heterogeneity regarding dietary nitrate intake in these populations, which may be attributed, in part, to a lack of publicly accessible data on nitrate and nitrite content in foods. Importantly, several efforts aimed at expanding the available data on dietary nitrate and nitrite content in a variety of foods are currently underway. The Bondonno group has created and maintained a reference database of nitrate content in plant- and animal-based foods with data extracted from studies conducted in several countries [[96], [97], [98]]. This robust resource is accessible to interested collaborators upon request. In the United States, the Department of Agriculture is currently partnering with the National Institutes of Health Office of Dietary Supplements to develop a special interest database featuring data on nitrate and nitrite content in a variety of foods, including infant formula and infant foods, and dietary supplements. This database is currently under development. In the future, information available from these databases will continue to improve the specificity that links plant sources of dietary nitrate with health benefits and risks.

On the clinical side, many systematic reviews and meta-analyses on the effectiveness of nitrate supplementation for cardiovascular health outcomes have been published in the past decade. Table 2 [[99], [100], [101], [102], [103]] summarizes the results of meta-analyses published in the last 10 y. These meta-analyses included, on average, 24 studies and consistently indicated that nitrate supplementation induces a significant blood pressure-lowering effect, especially for systolic blood pressure. Nonetheless, some important limitations impacting the results of the meta-analyses included in Table 2 must be noted: studies on nitrate supplementation are largely heterogeneous, which impacts the quality of the evidence and limits the pooling analysis. Furthermore, most trials were conducted with healthy participants. For this reason, the effectiveness of nitrate supplementation for those presenting with cardiovascular disease risk factors such as hypertension and hypercholesterolemia remains unclear, although evidence suggests that nitrate improves vascular function, stabilizes atherosclerotic plaques and decreases cardiac fibrosis in humans and animal models [76,84,104,105]. Finally, most participants included in studies on nitrate supplementation were male. Questions remain regarding optimal dosage, the role of physiological effect modifiers of dietary nitrate intake, and the target population.

**TABLE 2:** Study characteristics and results of meta-analyses on the effect of nitrate supplementation on outcomes related to cardiovascular disease risk factors

First author	Year	Number of studies (total number of participants)	Study population	Duration range	Nitrate source	Dose range (mg per dose)	Main outcomes	Results
M. Siervo [99]	2013	16 (254)	Adults	2 h to 15 d	Nitrate salt or beetroot juice	157–1488	Blood pressure	SBP: −4.4 mmHg (*P* < 0.001); DBP: −1.1 mmHg (*P* = 0.06)
J. Lara [100]	2016	12 (246)	Adults	90 min to 28 d	Nitrate salt or beetroot supplementation	68–1488	Vascular function parameters	Endothelial function: 0.4 (*P* < 0.001)
A. W. Ashor [101]	2017	13 (325)	Adults	1 to 6 wk	Nitrate salt or beetroot juice	322–620	Blood pressure	SBP: −4.1 mmHg (*P* < 0.001); DBP: −2.0 mmHg (*P* < 0.001)
J. K. Jackson [106]	2018	34 (813)	Adults	2 h to 70 d	Nitrate salt or dietary sources	55–1490	Blood pressure	SBP: −4.8 mmHg (*P* < 0.0001); DBP: −1.7 mmHg (*P* = 0.001)
D. Li [107]	2020	47 (1101)	Adults	3 to 168 d	Nitrate salt or dietary sources	150–1000	Blood pressure	SBP: −2.9 mmHg (*P* < 0.001); DBP: −1.5 mmHg (*P* < 0.001)
L. S. Bahrami [108]	2021	27 (765)	Adults	1 h to 6 wk	Beetroot supplementation	70–1500	Cardiovascular disease risk factors	SBP: −0.7 mmHg (*P* = 0.3); DBP: −1.3 (*P* = 0.06); HR: 8.6 bpm (*P* = 0.08); AIX: −3.3% (*P* = 0.1); FMD: 0.6% (*P* = 0.002)
Y. He [102]	2021	22 (372)	Older adults (>60 y old)	45 min to 4 wk	Nitrate salt or beetroot juice	25–840	Blood pressure	SBP: −3.9 mmHg (*P* < 0.001); DBP: −2.6 mmHg (*P* < 0.005)
Y. Zhang [103]	2023	19 (1069)	Adults	1 d to 4 wk	Nitrate salt or dietary sources	248–1165	Blood pressure	Healthy individuals: SBP: −2.42 mmHg (*P* = 0.01); DBP: −0.58 mmHg (*P* = 0.36) Hypertensive individuals: SBP: −0.82 mmHg (*P* = 0.35); DBP: −0.03 mmHg (*P* = 0.97)

Results are expressed as mean/standardized mean differences. All meta-analyses included in this table utilized randomized controlled trials and/or placebo-controlled trials and a significance level of 5%.

Abbreviations: AIX, augmentation index; AOS, arterial oxygen saturation; DBP, diastolic blood pressure HR, heart rate; SBP, systolic blood pressure.

Some of the meta-analyses have also explored the relationship between nitrate dose and physiological response through meta-regression and subgroup analyses. Jackson et al. [106] determined that nitrate dose was associated with resting systolic blood pressure (*P* = 0.031), but not with resting diastolic blood pressure (*P* = 0.315). They also determined that nitrate concentrations exceeding 650 mg/dose produced the greatest resting blood pressure reduction (systolic blood pressure: −10.45 mmHg, *P* < 0.0001; diastolic blood pressure: −6.31 mmHg, *P* = 0.045), but concentrations as low as 130–259 mg/dose were sufficient to produce significant results (systolic blood pressure: −5.52 mmHg, *P* = 0.007; diastolic blood pressure: −2.62 mmHg, *P* = 0.002). Contrastingly, Li et al. [107] reported that interventions including less than 445 mg/d had a greater lowering effect on systolic blood pressure than those utilizing 445 mg or more per day (*P* = 0.04). For diastolic blood pressure, this threshold was 460.5 mg per d (*P* = 0.04). In addition, the authors did not observe a dose-response relationship for systolic blood pressure but did observe an inverse linear relationship between nitrate dose and diastolic blood pressure (i.e., higher doses had smaller effects; *P* = 0.01). More recently, Bahrami et al. [108] concluded that diastolic blood pressure (*P* = 0.03), but not systolic blood pressure (*P* = 0.1), was associated with nitrate dose when analyzing the effects of beetroot supplementation. The lack of agreement between meta-analyses indicates that determining optimal recommendations for dietary nitrate to improve cardiovascular health remains challenging. The presence of unmeasured confounders (for example, oral microbiota nitrate-reducing capacity) as well as different methodological approaches and heterogeneity between studies may explain this disparity. For instance, most meta-analyses included studies of widely varying duration, which may be an important effect modifier. In fact, subgroup analyses showed that acute and shorter (<7 d) interventions are more effective at reducing blood pressure than longer interventions [106,108]. Furthermore, the lack of assessment of habitual dietary nitrate intake in clinical studies utilizing nitrate supplementation is another important confounder that may affect responsiveness to the intervention because of a possible ceiling effect. Overall, there is a clear need for more specific, well-defined meta-analyses evaluating the dose-response relationship between nitrate supplementation and physiological effects before we can establish a therapeutic dose range with adequate degree of confidence.

The evidence presented in Table 2 illustrates the difficulty in translating epidemiological findings to clinical settings. This may be explained by a myriad of factors that affect NOS-dependent and -independent pathways simultaneously. Perhaps one of the most important of these factors is age. Aging is associated with an increase in systolic blood pressure. This results from a variety of age-related physiological changes, such as increased reactive oxygen species burden and impaired NOS activity. Capper et al. [109] recently demonstrated that older adults (60–75 y old) had significantly lower plasma nitrite levels and required higher dietary nitrate concentrations to achieve blood pressure lowering relative to young adults (18–35 y old). The lower plasma nitrite concentrations in these older participants suggest potential mechanistic differences in the production of NO from dietary nitrate in young and older populations. Indeed, several aging-related factors also interfere with the NOS-independent pathway. For instance, older adults may have a lesser capacity for reducing nitrate to nitrite because of changes in the oral microbiome and lowered saliva production, decreasing the available nitrate and nitrite in this population, ultimately impairing NO homeostasis. The nitrate-reducing capacity of the oral microbiome is a primary contributor to systemic NO homeostasis and has been suggested as a potential pre- or probiotic targets to ameliorate age-induced impairments in cardiovascular and cognitive health. [110]. Other aging-related factors that interfere with the NOS-independent pathway include reduced gastric acid secretion or hypochlorhydria [111], increased proton-pump inhibitor usage [112], and decreased ascorbic acid bioavailability [113]. In this way, although older adults may be more dependent on the NOS-independent pathway to maintain systemic NO homeostasis, the aging process itself impairs their ability to respond to nitrate supplementation. Understanding these effect modifiers is important for the development of strategies to increase the effectiveness of dietary nitrate for individuals to whom supplementation is clinically relevant, such as older adults.

Sex differences represent another important modifier of the NO axis. Premenopausal females may have greater basal eNOS activity because of the effect of estradiol on eNOS expression [114], with a subsequent decline in endothelial function because of the loss of ovarian estradiol in menopause [115]. Reduced estradiol could alter the endothelial redox balance, thereby increasing oxidative stress and impairing endothelial function. Another sex difference is observed in the ability of the oral microbiome to reduce nitrate to nitrite. Kapil et al. [116] showed that females have a greater nitrate-reducing capacity compared with males despite a similar oral microbiome composition. Although the study was not designed to ascertain the cause of this observation, the authors hypothesize that female sex hormones may play a role in modulating bacterial reductase activity, drawing a parallel with estrogen-mediated regulation of eNOS expression [117,118]. Thus, intrinsic sex differences may play a role in an individual’s ability to respond to dietary nitrate.

Other relevant confounding factors affecting NOS-dependent and -independent pathways include antiseptic mouthwash use, thiocyanates from cruciferous vegetables, cyanide from cigarette smoke, sulfate in drinking water, and dietary lipid consumption. Although antiseptic mouthwash disrupts the oral microbiome necessary for nitrate reduction [47,119,120], thiocyanate, sulfate, and cyanide can decrease nitrate concentration in the saliva by blocking salivary ion transporters (for example, natrium-iodide symporter, chloride channel, and sialin) involved in nitrate uptake from blood [[121], [122], [123], [124]]. Finally, dietary lipids can increase NO auto-oxidation in plasma, thereby decreasing its bioavailability [35,125]. Identification of such factors provides insights that may explain the variable responses to nitrate and nitrite supplementation in clinical trials [35]. In this way, studies investigating the effect these interventions must also consider individual factors such as smoking status, usual dietary habits, and oral health to allow for appropriate multivariate and stratification analyses. Nonetheless, the body of evidence for the physiological benefits of nitrate for cardiovascular outcomes is persuasive. In this context, plant food sources of dietary nitrate may add to the arsenal of strategies to improve the health status of a population.

## Risks associated with dietary nitrate: context matters

As mentioned in the previous section, the 3 different dietary nitrate exposure routes–drinking water, animal-based foods (especially cured and processed meats), and vegetables and fruits–are associated with different health risks [126,127]. This distinction is paramount for the consideration of dietary nitrate specifically from plant-based food sources as a conditionally essential nutrient. Table 3 [[128], [129], [130], [131], [132], [133], [134], [135], [136], [137], [138], [139], [140]] presents meta-analyses on exposure to nitrate and nitrite and health risks published in the last 10 y. These meta-analyses included, on average, 23 studies that estimated nitrate and nitrite exposure using dietary assessment tools (for example, food frequency questionnaires) and food composition databases.

**TABLE 3:** Study characteristics and meta-analyses on the effect of nitrate and nitrite exposure on various cancer and fetal development defects risk

First author	Year	Number of studies	Study population	Exposure source^1^	Main outcomes	Nitrate exposure range	Nitrate results	Nitrite exposure range	Nitrite results
P. Song [128]	2015	49	Adults	Food sources and drinking water	Gastric cancer	66.4–220 mg/d	Gastric cancer risk: RR: 0.80 (95% CI: 0.69, 0.93)	0.1 mg/d	Gastric cancer risk: RR: 1.31 (95% CI: 1.13, 1.52)
Z. Bahadoran [129]	2015	15	Adults	Food sources and drinking water	Thyroid cancer	17.4–507 mg/d	RR: 1.36 (95% CI: 0.67, 2.75)	0.6–1.9 mg/d	RR: 1.48 (95% CI: 1.09, 2.02)
L. Xie [130]	2016	62	Adults	Food sources and drinking water	Site-specific cancers	10 mg/d	Gastric cancer risk: RR: 0.78 (95% CI: 0.67, 0.91)	0.5 mg/d	Adult glioma (RR: 1.21; 95% CI: 1.03, 1.42) and thyroid (RR: 1.52; 95% CI: 1.12, 2.05) cancer risk
N. R. Kakavandi [131]	2018	5	Adults	Food sources and drinking water	Neural tube defects	0–7 mg/d	RR: 1.33 (95% CI: 0.89, 1.99)	N/A	N/A
M. Yu [132]	2020	12	Adults	Food sources and drinking water	Non-Hodgkin lymphoma	NR^2^	OR: 1.02 (95% CI: 0.94, 1.10)	NR^2^	OR: 1.37 (95% CI: 1.14, 1.65)
M. S. SeyyedSalehi [133]	2021	10	Adults	Food sources and drinking water	Bladder cancer	NR^2^	OR: 1.06 (95% CI: 0.98, 1.15)	NR^2^	OR: 1.05 (95% CI: 0.98, 1.13)
A. Khodavandi [134]	2021	3	Adults	Food sources and non-food contaminants	Ovarian cancer	NR^2^	Non-food contaminant: RR: 1.36 (95% CI: 1.02, 1.80)	NR^2^	Non-food contaminant: RR: 1.07 (95% CI: 0.96, 1.16)
F. Hosseini [135]	2021	15	Adults	Food sources and drinking water	Colorectal cancer	NR^2^	Pooled HR: 1.04 (95% CI: 0.92, 1.19)	NR^2^	Pooled HR: 1.07 (95% CI: 0.95, 1.21)
K. S. Abasse [136]	2022	41	Adults	Food sources and drinking water	13 site-specific cancers	0–720 mg/d	Decreased risk of kidney (*P* = 0.002) and bladder (*P* = 0.008) cancers	0–35 mg/d	Increased risk of bladder (*P* = 0.056) and stomach (*P* = 0.000) cancers; decreased risk of pancreatic cancer (*P* = 0.007)
M. Nader [137]	2022	15	Adults	Meat products	Carcinogenic risk	95 mg/L	No identified risk (HRI = 0.0050)	55 mg/L	No identified risk (HRI = 0.1541)
A. Arafa [138]	2022	5	Adults	Drinking water	Bladder cancer	0.20–4.59 mg/d	OR: 0.98 (95% CI: 0.60, 1.57)	N/A	N/A
R. Picetti [139]	2022	60	Adults	Drinking water	Gastrointestinal, genitourinary, hematologic, and neurologic cancers	0.02–44.2 mg/L	Increased risk of gastric cancer (OR: 1.91, 95% CI: 1.09, 3.33) per 10-mg/L increment in nitrate exposure	N/A	N/A
N. R. Kakavandi [140]	2022	10	Adults	Food sources and drinking water	Heart defect risk in pre-term infants related to maternal intake of nitrate	1–8 mg/d	RR: 1.03 (95% CI: 1.00, 1.05; *P* = 0.400)	N/A	N/A

Abbreviations: CI, confidence interval; HR, hazard ratio; HRI, Health Risk Index; N/A, not applicable; NR, not reported; OR, odds ratio; RR, risk ratio.

1 Food sources include plant-based and animal-based sources.

2 Fixed effects estimate modeling in this study did not describe the quantitative range for low and high categories of nitrate and nitrite.

**FIGURE 2:**
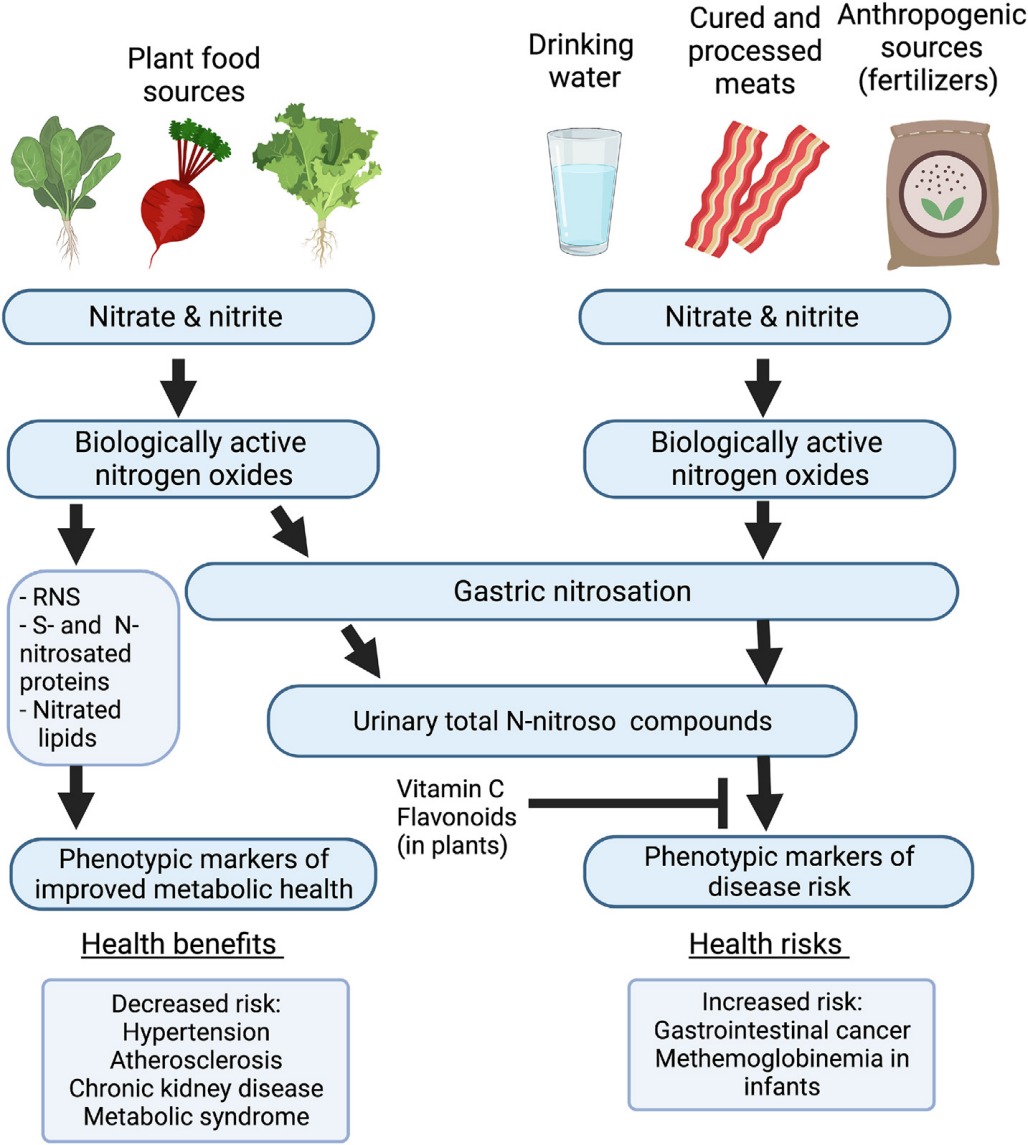
Different sources of dietary inorganic nitrate and nitrite have different benefits and risks. The health effects of nitrate from plant foods should be evaluated separately from those associated with nitrate from non-plant foods. This contextual relationship underscores the need for food-based dietary guidance for the consideration of nitrate from plant foods as a conditionally essential nutrient. Created with BioRender.com. RNS, reactive nitrogen species.

Although historically used in medical therapeutics for cardiovascular conditions, nitrate and nitrite experienced a “fall from grace” in the 1940s and 1950s when these chemicals became associated with methemoglobinemia risk in children [141] and were shown to contribute to nitrosamine formation and carcinogenesis [142]. Nitrosamines are produced via the reaction of nitrite and secondary amines in acidic conditions. The consumption of cured and processed meats, which typically contain nitrite or nitrate as preservatives, was then found to be of specific cancer types in many prospective epidemiological studies [143,144]. As organized in Table 3, recent meta-analyses evaluating the relationship of dietary nitrate and nitrite intakes with several cancer types reported an association between moderate-to-high nitrite intake and increased risk of thyroid, gastric, and adult glioma cancers [[128], [129], [130]]. However, these same studies reported no association between nitrate intake and cancer risk; in fact, some studies indicated that moderate-to-high nitrate intake was associated with a decrease in gastric cancer risk [128,130,145]. This strongly indicates that the health effects of dietary nitrate and nitrite are separate and dependent on the context in which they are consumed. Plant foods comprise a myriad of components that may confer protection against the aforementioned health risks [146] and also potentiate the beneficial effects of nitrate and nitrite (Figure 2). For instance, vitamin C can improve the effects of dietary nitrate on blood lipids [147] and vascular function [148], and flavan-3-ols add to inorganic nitrate effects on endothelial and vascular function [149,150]. In sum, although it is prudent to avoid excessive consumption of cured meats, the consumption of plant-based dietary nitrate should not be blindly categorized in the same way.

In drinking water, nitrate exists as a pollutant from human and animal waste and fertilizers. Thus, regulatory limits such as those imposed by the United States Environmental Protection Agency (<10 mg/L) are important to limit environmental pollution. In addition to the aforementioned cancer risk in adults, perceived risks regarding methemoglobinemia in infants (or “blue baby syndrome”) have also driven regulatory actions from several agencies, including the World Health Organization (WHO). The WHO has set the Acceptable Daily Intake for nitrate and nitrite at 222 and 4.6 mg, respectively, for an average 60-kg individual. For drinking water, the WHO has set the Maximum Contaminant Level for nitrate and nitrite at 50 and 1 mg/L, respectively [151]. The International Agency for Research on Cancer concluded that “ingested nitrate or nitrite under conditions that result in endogenous nitrosation is probably carcinogenic to humans (Group 2A)” [152]. This conclusion is based on human evidence regarding nitrite in food and gastric cancer risk, and animal evidence regarding nitrite with or without the addition of amines and amides. The same report concludes that there is insufficient evidence to classify nitrate in food or in drinking water as carcinogenic [152]. A workshop held at the National Institute of Heart, Lung, and Blood Institute concluded that nitrate consumed through vegetables in the diet are associated with cardiovascular protection, whereas nitrate in drinking water or under conditions that enhance gastric N-nitroso compound formation should be evaluated separately [146]. Finally, a recent panel of experts convened by the International Olympic Committee have concluded that dietary nitrate is an ergogenic aid and recommend that individuals seeking to use nitrate as such should increase their intake primarily via plant food sources (for example, beetroot juice and vegetables) [153,154].

Taken together, these positions further support the distinction between different routes of exposure to nitrate (dietary nitrate from plant food sources compared with nitrite salt in cured and processed meats) and health risks and benefits. Furthermore, it is important to note that population exposure to dietary nitrate is highly variable and underestimated. Hord et al. [7] demonstrated that 2 servings of high-nitrate vegetables can exceed the WHO Acceptable Daily Intake. Keller et al. [155] modeled dietary scenarios using foods and supplements and observed that an individual could consume as much as 601 mg/d when taking 1dietary nitrate supplement and consuming a DASH dietary pattern.. In addition, they reported that one serving of beetroot juice could contain up to 444 mg of nitrate [155]. These data underline the need for review of the current WHO Acceptable Daily Intake in light of the contextual evidence presented herein and elsewhere [156].

## Conditional essentiality: from evidence to dietary guidance

Currently, evidence-based recommendations for dietary nitrate intake levels are few. A recent group of experts commenting on potential ergogenic benefits recommended consumption of 8–16 mmol (500–1000 mg) of nitrate acutely or 4–16 mmol (250–1000 mg) of nitrate daily from vegetables and vegetable juices based on clinical studies [153]. This suggested range of nitrate intake is higher than the typical estimates of 1.5–2 mmol (93–124 mg) daily for most populations except some notable dietary patterns such as Chinese and Japanese, which yield 3.6 mmol (220–230 mg) daily [155], as well as Mediterranean, which yields 19.4 mmol (∼1200 mg) per day [7]. However, it is important to note that different contexts are associated with different recommendations. In other words, clinical studies are conducted in a therapeutical context and, as such, typically employ pharmacological doses of a drug or supplement, whereas observational studies ascertain average intakes associated with habitual dietary consumption. Thus, efforts to define recommended intake levels of dietary nitrate from plant foods within specific contexts are imperative to translate epidemiological and clinical findings into dietary guidance.

As mentioned in the Introduction, the DRIs are intended for healthy populations [1], whereas Specialized Nutrient Requirements are intended for disease states in which nutrient requirements are altered because of pathophysiological processes [3]. In this context, experts have worked to articulate a new approach to define evidence-based, systematic guidelines for future DRIs to include characteristics of conditional essentiality linked to surrogate biomarkers of exposure and risk related to chronic disease endpoints [157]. These guidelines provide a necessary framework for understanding how nutrient recommendations may apply in specific situations. Considering the evidence presented herein, we contend that this guidance is highly relevant for dietary nitrate obtained from plant-based sources and therefore nominate it for consideration as a conditionally essential nutrient employing the revised DRI guidelines.

Another pathway for proposing practice recommendations emerged recently with the publication of the first dietary bioactive guideline for flavan-3-ols [158]. Although the DRI process is overseen by the National Academies, this guideline was developed using a process outlined by the Academy of Nutrition and Dietetics. Of note, the recommendations are based on health benefits instead of avoiding deficiency, particularly those related to cardiovascular outcomes. In this context, we can draw a parallel between dietary nitrate from plant-based sources and dietary flavan-3-ols and their effects on cardiovascular health. Based on the body of evidence regarding dietary nitrate and cardiovascular outcomes, we believe that dietary nitrate from plant food sources is a relevant candidate for similar practice recommendations.

## Conclusion and future directions

The petition presented herein is built upon a wealth of evidence supporting the conditionally essential nature of dietary nitrate. Pathophysiological conditions that involve chronic inflammation and increased oxidative stress (for example, cardiovascular diseases) are associated with decreases in whole-body NO synthesis and plasma nitrite levels. This indicates that NO availability is impaired under these conditions. Dietary nitrate provides an exogenous input to NO production (Figure 1) with demonstrated cardiovascular benefits in both observational and interventional studies (TABLE 1, TABLE 2). Finally, risks associated with other sources of dietary nitrate and nitrite (processed meats and drinking water) are not evident with plant-based sources; instead, exposure to plant-based sources of dietary nitrate are associated with health benefits and cardiovascular protection. We, like other groups [126,159], understand that additional well-designed epidemiological studies will be necessary to clarify concerns about cancer risk related to dietary nitrate intake.

Our rationale recognizes the interindividual differences that act as potential confounders in studies investigating the role of inorganic nitrate in cardiovascular health. Future studies should be designed to assess specific effect modifiers of dietary nitrate bioavailability (for example, age, sex, nitrate-reducing capacity of the oral microbiome, co-morbid conditions, and dietary and pharmacological agents affecting NO bioavailability, etc.) [159]. These assessments will be clinically useful when determining which individuals are most likely to benefit from consuming more nitrate-rich plant foods.

Although the potential health benefits and risks of dietary nitrate and nitrite intake presents a conundrum for dietary recommendations [155,156], these opposing risks are overcome with careful consideration of dietary nitrate from plant-based foods as a conditionally essential nutrient. Moreover, these opposing risks are addressed by providing recommendations based on plant food sources of nitrate that confer demonstrated cardiovascular benefits. Food-based dietary guidelines as championed by the Food and Agriculture Organization [160] and American Institute for Cancer Research [161] are an optimal vehicle to promote the consumption of such foods in a context-informed and evidence-supported manner. Particularly, food-based guidelines can overcome practical challenges associated with the considerable variability in the nitrate content of foods. Like other plant compounds (for example, polyphenols), variability is expected because of the myriad of factors that influence the biological processes involved in synthesizing these compounds in plants. Nonetheless, food patterns such as the Mediterranean and DASH diets [7] have demonstrated that it is possible to consume high amounts of nitrate by choosing appropriate foods. Furthermore, dietary guidelines, including the DRIs, consider that an individual should meet these recommendations within their usual diet. This means that one is not required to consume a fixed amount of a micronutrient each day, but rather that their usual diet provides, on average, an adequate amount of this nutrient.

The complexity of human nutrition requires a nuanced and contextualized approach informed by evidence. The case of dietary nitrate is a perfect example of this complexity in which a careful examination of available evidence is merited. In conclusion, the roles of plant-based dietary nitrate in NO homeostasis and cardiovascular health represent cogent arguments for its consideration as a conditionally essential nutrient.

### Author contributions

The authors’ responsibilities were as follows – ACdCP-L, RMD, MCP, HVMN, KV, NGH: conceptualized research; ACdCP-L, NGH: wrote the paper; ACdCP-L, RMD, MCP, HVMN, KV, NGH, FH: reviewed the manuscript; NGH: had primary responsibility for final content; and all authors: read and approved the final manuscript.

### Conflict of interest

The authors report no conflicts of interest.

### Funding

This work was supported by OU Health Harold Hamm Diabetes Center and the Celia Strickland Austin and G. Kenneth Austin III Professorship in Public Health and Human Sciences at Oregon State University. Supporting sources had no involvement in this publication.
